# Identification of avian W-linked contigs by short-read sequencing

**DOI:** 10.1186/1471-2164-13-183

**Published:** 2012-05-14

**Authors:** Nancy Chen, Daniel W Bellott, David C Page, Andrew G Clark

**Affiliations:** 1 Department of Ecology and Evolutionary Biology, Cornell University, Ithaca, New York, USA; 2 Cornell Laboratory of Ornithology, 159 Sapsucker Woods Road, Ithaca, New York, USA; 3 Howard Hughes Medical Institute, Whitehead Institute, and Department of Biology, Massachusetts Institute of Technology, 9 Cambridge Center, Cambridge, Massachusetts, USA; 4 Department of Molecular Biology and Genetics, Cornell University, Ithaca, New York, USA

**Keywords:** Sex chromosomes, Next-generation sequencing

## Abstract

**Background:**

The female-specific W chromosomes and male-specific Y chromosomes have proven difficult to assemble with whole-genome shotgun methods, creating a demand for new approaches to identify sequence contigs specific to these sex chromosomes. Here, we develop and apply a novel method for identifying sequences that are W-specific.

**Results:**

Using the Illumina Genome Analyzer, we generated sequence reads from a male domestic chicken (ZZ) and mapped them to the existing female (ZW) genome sequence. This method allowed us to identify segments of the female genome that are underrepresented in the male genome and are therefore likely to be female specific. We developed a Bayesian classifier to automate the calling of W-linked contigs and successfully identified more than 60 novel W-specific sequences.

**Conclusions:**

Our classifier can be applied to improve heterogametic whole-genome shotgun assemblies of the W or Y chromosome of any organism. This study greatly improves our knowledge of the W chromosome and will enhance future studies of avian sex determination and sex chromosome evolution.

## Background

While whole-genome shotgun and short-read assemblies are rather effective at reconstructing single-copy euchromatic genes, repetitive regions remain a major challenge. Short-read sequencing eliminates issues related to low cloning efficiency of interspersed repeats, but the assembly process remains problematic for both repeats and segmental duplications, as high sequence homogeneity among copies of a given repeat or duplication limit the potential to reconstruct sequence order [1,2]. The inability to assemble repetitive regions can also pose difficulties for reconstructing large scaffolds from contigs [3], and the resulting gene fragmentation complicates gene assembly and annotation [2]. The assembly of repeats and duplications therefore remains a major challenge in genome sequencing and is only possible by focused and concerted efforts [4,5].

In species with chromosomal sex determination, the male-specific Y (in species with XX/XY sex determination) and female-specific W chromosomes (in species with ZZ/ZW sex determination) present special challenges to whole genome shotgun assembly. Sex-specific chromosomes are enriched for interspersed repeats and segmental duplications, on which whole genome shotgun methods perform poorly [5]. The absence of crossing-over outside the pseudoautosomal region makes it impossible to take advantage of the genetic map for scaffolding the assembly [6]. An additional hindrance is the lower sequence coverage of the sex chromosomes when sequencing heterogametic individuals, which reduces the average length of assembled contigs. Sex chromosomes receive half the coverage of autosomes when sequencing heterogametic individuals (the strategy used for chicken and turkey), and just a quarter of the autosomal coverage if sequencing a 50:50 mix of heterogametic and homogametic individuals (the strategy adopted for *Drosophila melanogaster*). Even in organisms like *Drosophila melanogaster*, where the quality of the whole genome shotgun assembly is extremely high, the Y chromosome remains a collection of unassembled contigs [7-9]. In the case of humans and chimpanzee, the Y chromosome assemblies are nearly complete, because these were sequenced by a painstaking BAC-by-BAC effort [5,10].

There is considerable interest in assembling the female-specific avian W chromosome, not only to expand our understanding of sex-determination mechanisms, but also to address many questions about sex chromosome evolution. The exact mechanism of avian sex determination remains controversial: though the Z-linked *DMRT1* gene is required for testis development (which is consistent with the Z dosage hypothesis), female sex determination may still involve a dominant, W-linked gene (analogous to Y-linked dominant sex determination in mammals) [11,12]. More information about the W chromosome will contribute to our understanding of the evolution of female heterogamety as well as the dynamics of sex chromosome degradation and differentiation [13].

The chicken genome, which contains 38 autosomes and a pair of sex chromosomes, was sequenced in 2004 from a single female Red Junglefowl [14]. About 70% of the heterochromatic chicken W chromosome consists of *XhoI*-, *EcoRI*-, and *SspI*-family repetitive sequences, and some known genes on the W are tandemly duplicated (*e.g., Wpkci*[15]), leaving an estimated 10–15 Mb of non-redundant sequence [16]. The chicken genome was sequenced to 6.6x coverage and assembled from whole-genome shotgun reads, as well as plasmid, fosmid, and bacterial artificial chromosome (BAC)-end read pairs [14]. Of the 1.05 Gb of assembled sequence, only 933 Mb were anchored to a specific chromosome, leaving 121 Mb in unmapped sequence fragments, collectively called chrUn [14]. Assembly of the W chromosome is especially poor: only 0.5% of the W (based on its estimated size of 50–55 Mb) has been successfully mapped. To date, only a handful of genes have been identified on the W: *CHD1W*[17], *ATP5A1W*[18], *ASW/Wpkci/HINT1W*[15,19],* SPINW*[20], *SMAD2*[16], *UBAP2W/ADO12W*[21], *ZNF532W*[22], *ZFRW*[22], *MIER3W, hnRNPKW*[23], *SSC2W/NIPBLW*, and *KCMFW* (first identified in Build 2.1 and then cited by [23]).

Given the challenges in producing an assembly of the Y and W chromosomes by traditional shotgun-sequencing methods, new tools are required to identify sex-specific sequences generated by heterogametic shotgun sequencing projects. Here, we adapt a method devised by Carvalho and colleagues (unpublished; the original approach was aimed toward discovering Y-linked contigs in *Drosophila*) and identify female-specific sequences by contrasting male-derived, short-read shotgun genomic sequences and unmapped sequence fragments (chrUn) from the female-derived chicken genome. This method relies on the fact that the W chromosome is female-limited. By sequencing the genome of the homogametic sex (in our case, the ZZ male) to high depth and aligning the reads to the genome of the heterogametic sex (the ZW female), we were able to identify regions of the genome that are underrepresented in males and are therefore likely to be female-specific.

## Results

### Conceptual framework

**Figure 1:**
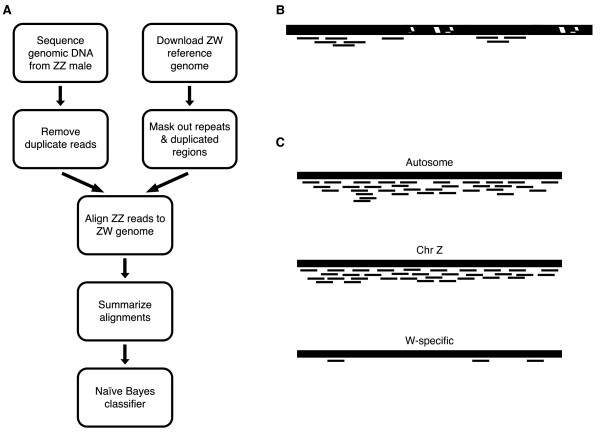
**A novel method of identifying W-specific contigs.** (**A**) The steps in our classification procedure. (**B**) Alignment results were summarized by two statistics: coverage and read depth. If a contig consists of unique sequence (solid black) and masked repetitive regions (hatched), then coverage is the proportion of unique sequence covered by a read. Read depth is the number of reads divided by the total possible locations to which a read could map. (**C**) Predicted alignment results. Each W-specific contig should have very few male-derived reads uniquely aligning to it.

Because avian males carry two Z chromosomes, the male genome should not contain any sequence that is found exclusively on the W chromosome. Thus, when mapping reads generated from a male (ZZ) back to the shotgun genome assembly generated from a female (ZW), very few, if any, reads should uniquely map to segments of the female ZW genome that are W-specific. In particular, evidence that unmapped contigs from the ZW female are likely to be W-specific derives from their under-recruitment of matches to the reads from ZZ males (see overview of method in Figure1). This method is similar to the read depth approaches for detecting copy number variants, which assume a Poisson distribution in mapping depth and therefore detect duplications and deletions by searching for regions with significantly higher or lower read depth [24,25]. Our pipeline relies on the subtraction of the male genome from the female genome and tests for lower read depth on a contig-by-contig basis. We summarize alignment results for each contig using both the number of unmasked bases covered by a read (coverage) and a normalized measure of total number of reads aligned (read depth; Figure1B). Both measures should be near zero for W-specific contigs but not for autosomal or Z-linked contigs (Figure1C).

### W-specific contigs have distinct coverages and read depths

**Figure 2:**
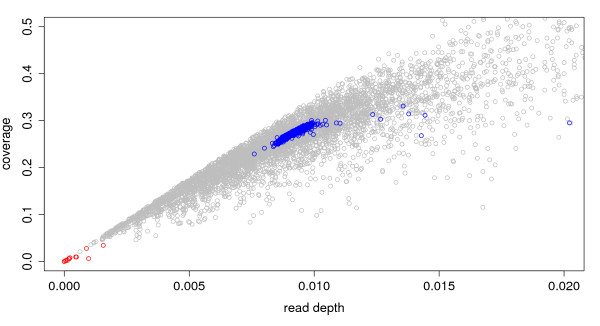
**Discrimination between W and non-W contigs based on read depth and coverage for each contig.** Known W contigs are shown in red, known Z or autosomal contigs are in blue, and unmapped contigs are in gray. Note that the W contigs (red dots) exhibit very low alignment and read depth to male-derived sequences. The W contigs form a distinct cluster from the autosomal or Z contigs. The goal is to classify all the unmapped contigs (gray dots) into one of two classes: W or non-W.

We generated roughly 10 million reads from a ZZ individual and mapped them to the unique regions of the ZW genome. As predicted, the previously-mapped W-linked contigs had significantly fewer uniquely aligned reads relative to known autosomal and Z-linked contigs (Figure2). The known W-contigs have coverage and read depth values near zero: 95% bootstrap CI for coverage is (0, 0.083) and for read depth (2.24x10^−6^, 0.0139). This was expected because sequences derived from a male genome are not expected to map to W-linked contigs. In contrast, male-derived sequences readily align to known autosomal and Z-linked contigs. Autosomal/Z-linked contigs have non-zero read depths and coverages: the 95% bootstrap CI for coverage (0.256, 0.293) and for read depth (0.00862, 0.00992) both are positive. Thus W contigs have significantly different coverage and read depth values than autosomal/Z-linked contigs (p = 0).

### Contig length influences alignment results

**Figure 3:**
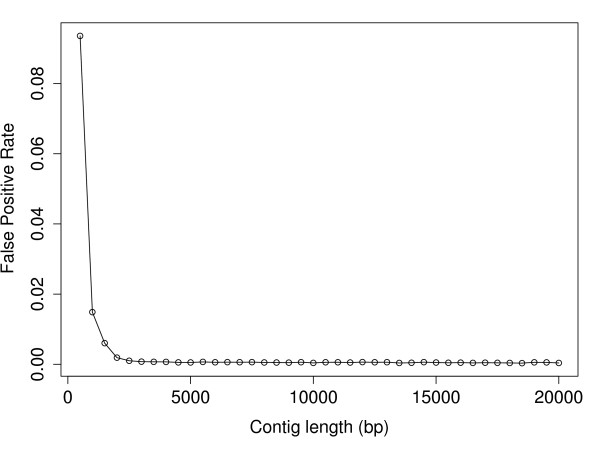
**False positive rate as a function of contig length.** Here the false positive rate refers to the fraction of known autosomal or Z-linked contigs with coverage less than 0.10 (the mean 100^th^ quantile for coverage of known W-specific contigs from the bootstrap replicates).

Due to the stochasticity of the sequencing method, the length of the contig may affect the distribution of hits along the contig and therefore our prior expectations of both coverage and read depth. We simulated several genomes, each with contigs of a different length. Once contig length decreased to 1,500 bp or less, the probability that an autosomal or Z-linked contig would be misclassified as a W-specific contig increased exponentially (Figure3). After stringent filtering, 57% of the remaining 6,905 unmapped contigs are of length 1,500 bp or less. It is therefore important to take contig length into consideration in the classification method. Not accounting for the fact that very short contigs have fewer hits regardless of class would greatly inflate the false positive rate of the classification approach.

### Evaluation of performance

**Figure 4:**
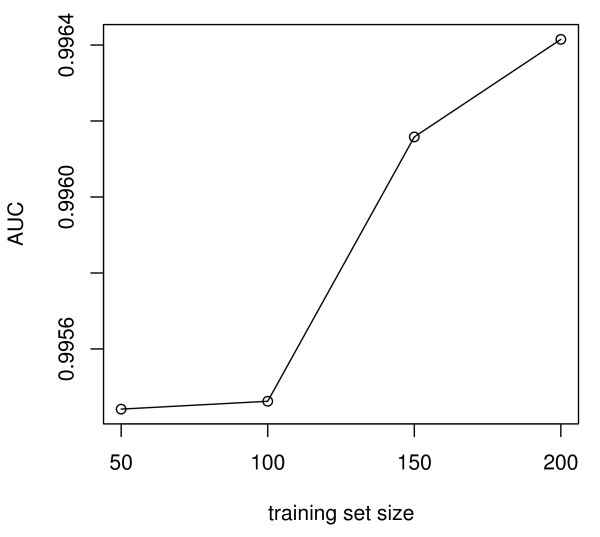
**Performance of the classifier as a function of number of contigs in the training set.** We ran the classifier with increasing numbers of contigs, from 50 W & 50 non-W contigs to 200 W & 200 non-W contigs. This was done by subsetting the mapped contigs 100 times: for each iteration, the set of training contigs was randomly selected, and the remainder used for validation. The mean AUC for each training set size is shown. AUC (area under the ROC curve) is a commonly used statistic for model comparison.

We developed a naïve Bayes classifier to determine which of the unmapped contigs are likely to be W-specific. A naïve Bayes classifier relies on a set of training data to estimate parameters for classification. Thus properties of the training set may significantly affect the performance of the classifier. We performed several different experiments to optimize the classifier. By running cross-validation tests with the previously mapped contigs, we investigated the effects of training set size, sample imbalance, and bin sizes of the feature distributions on the classifier. ROC curves were generated and the area under the curve calculated for all variations of the method. Increased training set size improved the performance of the classifier (Figure4). This result is not surprising: the more data used to estimate model parameters, the better the classifier performs. Sample imbalance occurs when there is unequal representation of different classes in a dataset. Imbalanced datasets can negatively impact the performance of machine learning algorithms. However, in our case, sample imbalance did not seem to be a problem: we ran the classifier with different ratios of non-W:W contigs (from 1:1 to 100:1) in the training set and found no significant differences in performance. Finally, we also tested different variations of the feature probability distributions. Evaluation of the different bin sizes for discretizing distributions of coverage and read depth found that the optimal bin size is 0.005.

**Figure 5:**
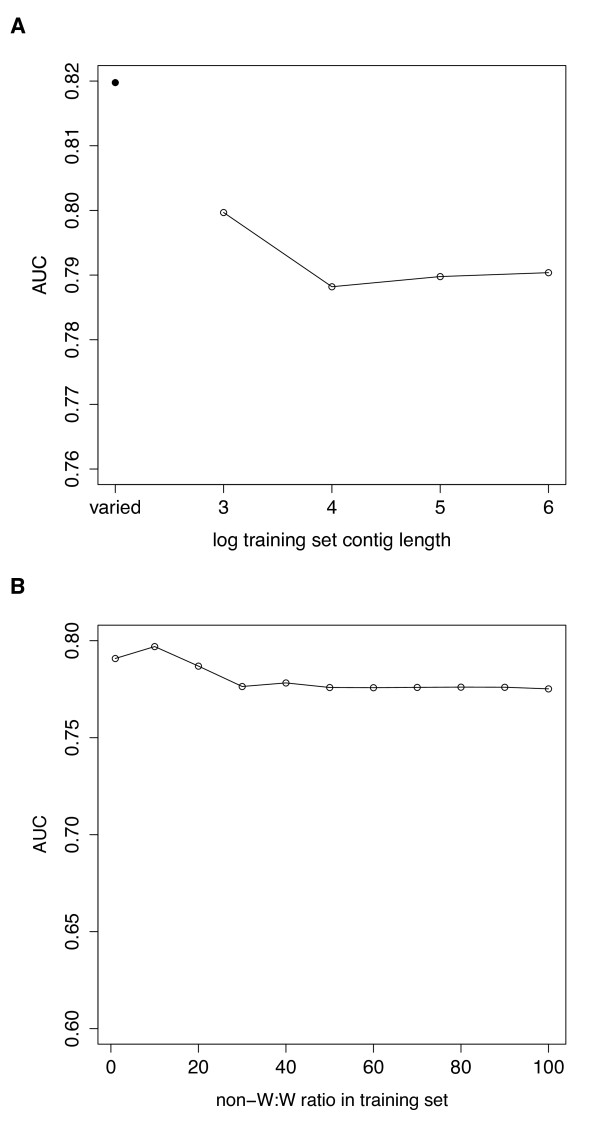
**Performance of the classifier as a function of contig length and training set composition.** Here the validation set consists of the confirmed chrUn contigs, and the training set is a subset of the set of mapped contigs. (**A**) Contig length matters. The open circles show results without conditioning on length; instead, the same validation set was classified using training sets with different contig lengths (1 kb - 1000 kb). For each contig length, we randomly selected 200 W & 200 non-W contigs for the training set. This was performed 100 times. The validation set contigs are short (average <1 kb in length), and the classifier performs better when shorter contigs are used for training. However, performance is maximized when we condition on length in the classifier (solid circle). Classifier performance is measured by mean AUC. (**B**) AUC for different ratios of non-W to W contigs in the training set. AUC increases for smaller non-W:W ratios.

After optimizing the classifier using known data, the next step was to evaluate the ability of the classifier to accurately predict novel W sequences. Our classifier identified 629 candidate W-specific contigs from the set of unmapped contigs. We have tested 315 contigs by PCR and confirmed 62 of them as female-specific (Additional file 1). Of these, we found female-specific markers on 51 of the 177 contigs that had a >95% posterior probability of being W-specific. We used these results to further evaluate the sensitivity and specificity of our method in independent data set tests. In these tests, the contigs of known location were used to train the data set, and performance of the classifier was evaluated using the PCR-confirmed set. A series of independent data set tests were used to test the effects of contig length and sample imbalance on classifier performance. Our simulations (see above) predicted that contig length should influence classification results. To test this prediction, we used contigs of varying sizes to train the classifier and compared performance on the same validation set of short (mostly 1 kb) contigs. Classifier performance decreased substantially when >10 kb contigs were used to train the classifier. Contig length does affect classification results, which explains why greater accuracy is achieved by conditioning on contig length (Figure5A). Unlike the results from our cross-validation tests, sample imbalance had more of an effect in these independent data set tests. Performance improves slightly when the non-W:W ratio is below 10 (Figure5B); therefore, severely unequal representation of the non-W and W classes affects the predictive performance of the classifier. However, sampling methods such as over-sampling the minority class (W) or under-sampling the majority class (non-W) can achieve better results. Overall, the classifier did not perform as well in the independent data set tests, most likely due to the high false positive rate that resulted from insufficient sequence coverage.

## Discussion

We present a framework to identify W-specific sequences in the chicken genome. The approach is generalizable to identify any genomic sequences that are present uniquely in one sex (*e.g.,* Y or W chromosomes within other animal species), and is potentially useful for characterizing the genomes of non-model organisms. Our method is based on the fact that sequences unique to the W chromosome are not present in the genome of a male. We mapped male-derived sequence fragments to the genome of a female and developed a naïve Bayes classifier using the alignment results (summarized by coverage and read depth). As predicted, contigs specific to the W chromosome had significantly lower coverages and read depths.

The accuracy of our method can be improved with deeper sequencing. Many of the false positive contigs probably had low coverages and read depths due to low sequence depth. We generated 367.2 Mbp of high quality sequence, which translates to only 0.45x coverage of the masked genome. It is therefore not surprising to find portions of the genome misleadingly underrepresented in the data set. At half this coverage, 40% of contigs of length 1 kb have very few reads aligning, making it more difficult to distinguish true female-specific contigs. However, this depth of sequencing was sufficient for proof of concept. We show that, even at low coverage, the approach was successful at identifying a focal set of candidate sequences for subsequent verification by targeted PCR.

Unlike traditional sequence mapping methods, our approach is not severely hindered by the lower sequence coverage of the W chromosome during shotgun sequencing of heterogametic individuals. While lower coverage results in W contigs that on average are shorter in length (and therefore more difficult to classify), we greatly improve performance by conditioning on contig length in the classification method. However, our method cannot fully overcome the challenges posed by repetitive regions. All interspersed repeats and segmental duplications were masked out of the genome before performing the alignments, thereby eliminating much of the W chromosome from consideration. It is possible to relax the stringency of the filtering step in further iterations of the classifier to identify euchromatic repeats that do not resemble genome typical repeats. Furthermore, this method cannot exhaustively find all non-repetitive W contigs – it can only detect unique regions specific to the W. Sequences in the pseudoautosomal region will produce the same read depth as autosomal regions, and recent gene duplication events may produce W-linked sequences with enough similarity to autosomal or Z-linked sequence to be represented in male genomes.

Because our method searches for regions in the male genome that are underrepresented in female-derived genome sequences, any male-specific deletions could lead to an inappropriate assignment of contigs to the W chromosome. Deletions in the White Leghorn genome compared to the Red Junglefowl genome are not an issue because all our PCR validations used males and females of the same species. Our method would classify a deletion in the White Leghorn genome as W-specific, but such a region would not show a female-specific amplification pattern in our PCR validation step. Misclassifications due to male-specific deletions can be detected by screening a larger set of individuals and by BAC screening and sequencing.

Despite the limitations of our approach, we were still able to identify more than 62 new W-specific contigs. Note that this number is an underestimate, as contigs that fail to produce a female-specific marker may still be located on the W chromosome. These new markers will greatly improve the assembly and annotation of the W chromosome. A more complete annotation of genes on the chicken W chromosome will accompany the BAC-based sequencing and assembly of the chromosome.

There is particular interest in fully annotating the avian W because the sex-determining mechanism in birds has yet to be completely characterized. *DMRT1* is known to be required for testis development [11], though studies on triploid and chimeric chickens suggest there may be a female-determining gene that interacts with a male-determining locus on the Z [26,27]. Evidence supporting the popular W-linked candidate, *HINTW*, is mixed: though *HINTW* is functionally different from its Z chromosome paralog [15], mis-expression of *HINTW* in male (ZZ) embryos resulted in normal testes development [26]. Further annotation of the W may unearth other candidate ovary-determining genes.

Sequence information of the W chromosome would benefit several different evolutionary studies besides avian sex determination, from sex chromosome evolution to sexual conflict and sex-biased mutation rate [12]. For example, birds are good subjects for the study of sex chromosome evolution because different bird groups exhibit parallel divergence of the W as well as variation in the degree of W chromosome degradation (from a largely undifferentiated state in ratites to a highly degenerate state in passerines) [13,28]. The scope for genetic conflict is increased in ZW species because the W is expressed in both sexes in the form of maternal effects, and the accumulation of sexually antagonistic maternal effect genes could contribute to the decay of the non-recombining W [29]. The W chromosome may be a magnet for female-specific fertility genes. Evolutionary theory indicates that male fertility genes are expected to be retained on the Y chromosome because they are free from the influence of selection in females [30,31]. By symmetry, this same evolutionary theory leads to the expectation that the W chromosome may concentrate genes that are uniquely necessary for female fertility [30,31]. Finally, ZW systems may be more appropriate than XY systems for studying sex-specific mutation rates: while higher mutation on the Y may be due to male-biased mutation or suppressed mutation on the X chromosome to minimize exposure of deleterious recessives in the hemizygote male, these hypotheses can be distinguished in ZW sex chromosomes [32].

The availability of more W-specific sequences also facilitates the development of additional sex-specific primers for unambiguous molecular sexing techniques. The ability to sex individuals is critical for answering several questions in evolution and ecology, and morphological identification of sex is often difficult in birds [33]. The commonly used universal primer sets for avian molecular sexing depend on length differences between *CHD-Z* and *CHD-W* introns [34-36], which may be problematic in certain species due to *CHD-Z* polymorphisms [37] and heteroduplex molecule formation [38]. Thus the new W-specific sequences identified here can help advance several different avenues of research.

## Conclusions

Here we describe a novel approach for identifying sequences specific to a heterogametic sex chromosome. We performed a proof-of-concept experiment by aligning shotgun sequence reads from a male (ZZ) chicken to the genome of a female (ZW) chicken, and our classifier successfully identified >60 confirmed novel W-specific contigs despite low coverage. We believe that our method is widely applicable and can benefit future genome assembly efforts. While there have been significant investments in lowering sequencing costs and increasing sequencing throughput, little investment has been made in techniques to cope with the limitations of whole-genome shotgun sequencing strategies, particularly the challenges specific to sex chromosomes: low coverage, resolution of interspersed repeats and segmental duplications, inability to map, etc. In addition, de novo assemblies generated using only next-generation sequencing technologies are especially prone to collapsing segmental duplications and large repeats [2]. The approach described here can quickly identify candidate W or Y chromosome markers, and these contigs can be extended by probing BAC libraries. A full assembly of the W chromosome still requires substantial BAC sequencing efforts, but this method can greatly facilitate the process of designing W-specific probes. A combination of our method with traditional BAC screening and sequencing would provide a powerful approach to assembling the W or Y chromosome in any organism.

## Methods

### Data generation

Genomic DNA was extracted from the blood of a White Leghorn rooster using the Qiagen DNeasy kit. We generated 10.5 million 36 bp reads using the Illumina Genome Analyzer (GA-IIx). Duplicate and low-complexity reads were removed before alignment, resulting in a total of 10.2 million unique and high quality reads. The sequence data generated in this study have been submitted to the NCBI Sequence Read Archive (http://www.ncbi.nlm.nih.gov/sra) under accession SRP008449.

We obtained chicken genome sequences (Build 2.1) and known W chromosome BAC sequences. The chicken genome assembly includes 18 scaffolds mapped to the W chromosome, and 1044 autosomal or Z-linked scaffolds. The 25,378 unmapped contigs (chrUn) had lengths ranging from 54 to 48,370 bp. Low complexity sequences and repeats were masked with RepeatMasker (http://ftp.genome.washington.edu/cgi-bin/RepeatMasker/). After removing segments less than 50 bp in length, this resulted in 920.7 Mbp of sequence and 20,069 unmapped contigs. However, because our method relies on the unique mapping of reads, any sequences that occur in multiple locations in the genome could lead to spurious results. Thus, more stringent filtering of the reference genome was required. We aligned the masked contigs to themselves in MUMMER [39] and masked any duplicate regions larger than 50 bp. After this more stringent filtering step, we were left with a total 823.7 Mbp of unique sequence, with 6,905 unmapped contigs.

Reads were aligned to the masked and filtered reference genome using MAQ [40]. We allowed some mismatches in the alignment process to account for sequence divergence between White Leghorn and Red Junglefowl [41]. Alignment results were summarized for each contig using two statistics: coverage and read depth (Figure1B). Here we define coverage as the fraction of unmasked bases in a contig that is covered by one or more reads. Read depth is the number of reads aligning to a contig, normalized by the total number of locations a read could align to that contig. Our measure of read depth is analogous to the widely used measure of gene expression, reads per kilobase of exon model per million mapped reads (RPKM). Because we used only one library, there was no reason to calculate RPKM, which standardizes among libraries.

### Confirmation of predictions

Because a large portion of the initial chicken W chromosome assembly was later discovered to be misassigned [14,42], we used genomic BLAST to ensure that the W contigs in our reference genome are representative of W-specific sequence. In addition, we confirmed any outliers in the initial W-specific set by comparing features of each W contig to features of the known set of autosomal and Z-linked contigs. We used 1000 bootstrap replicates to estimate confidence intervals of mean coverage and read depth for known autosomal or Z-linked contigs, which were then compared to the coverage and read depth values, respectively, of each putative W-specific contig.

Our method is based on the assumption that very few ZZ reads should align to W-specific contigs, which as a result should have significantly lower coverage and read depth compared to autosomal or Z-linked contigs (Figure1C). To confirm the predictions of our method, we compared the coverage and read depth for contigs of known location. We used nonparametric bootstrapping methods to determine whether known W and known autosomal or Z-linked contigs had different distributions of coverage and read depth. For each of the 1000 bootstrap replicates, we calculated the difference between the 100^th^ quantile of the W bootstrap distribution and the 0^th^ quantile of the non-W-specific bootstrap distribution. This difference should be positive if the distribution of coverage or read depth of autosomal/Z-linked contigs is distinctly greater than that of W-specific contigs.

### Simulations to determine effect of contig length

Because the length of unmapped contigs varied greatly (from 50 to 44,574 unmasked bp), we tested the effect of length by simulating genomes consisting of different-sized contigs. Contigs were sorted by length into 500 bp bins. We fragmented the mapped portion of the reference genome into contigs of length 500 bp, 1 kb, etc. For each fragmented genome, we redid the alignments and compared the distributions of coverage and read length for W- and non-W-specific contigs.

### Classification approach

We developed a naïve Bayes classifier to identify W-specific contigs. A naïve Bayes classifier uses a set of training data to calculate the probability that a given example belongs to a certain class based on a set of features. It simplifies the learning process by assuming that the features are independent, although in practice it performs well even if that assumption is violated. We will refer to each contig by its feature vector *X* = (*x*_*1*_, *x*_*2*_), where *x*_*1*_ is coverage and *x*_*2*_ is read depth. The goal is to find the class *C* that maximizes the likelihood: P(*X|C*) = P(*x*_*1*_,*x*_*2*_|*C*). *C* can be either W or non-W. Since we assume that *x*_*1*_ and *x*_*2*_ are conditionally independent, we can simplify this conditional probability to P(*X|C*) = P(*x*_*1*_|*C*)P(*x*_*2*_|*C*).

To account for the effect of contig length, we conditioned on length in the classification method as follows: given a contig *X* with length *L* (rounded to the nearest 500 bp), we rewrite the likelihood as P(*X|C,L*) = P(*x*_*1*_|*C,L*)P(*x*_*2*_|*C,L*). The training set therefore depends on the contig length: for contigs of length *L*, the training set consists of mapped contigs of length *L* (see length simulations above). The feature probability distributions P(*x*_*i*_|*C,L*) are estimated from the relative frequencies of the appropriate training set. Both the coverage and read depth distributions were discretized into bins of equal width. We tested several bin widths: 0.0005, 0.001, 0.005, 0.01, and 0.05. Thus P(*a* < *x*_*i*_ < *b|C,L*) is the frequency of contigs of class *C* with *a* < *x*_*i*_ < *b* in the genome with length *L* contigs + ϵ, where ϵ is close to zero. This small sample correction is necessary because zero probabilities cause information loss. The posterior probability that a given contig is W-specific is then:

(1) $$P(C\in W|X,L)=\frac{P(C\in W)P(X|C\in W,L)}{P(X|C\in W,L)+P(X|C\in nonW,L)}$$

### Performance

We assessed the performance of our test using Receiver Operating Characteristic (ROC) curves. ROC curves plot the true positive rate and false positive rate of a classifier over a range of threshold values, and the area under the curve (AUC) is a traditionally used statistic for model comparison. We generated ROC curves and calculated the AUC using the package ROCR in the R statistical package (http://www.r-project.org). A series of cross-validation tests using the previously-mapped contigs was used to fine-tune the bin sizes of classifier feature distributions and evaluate the effects of training set size and sample imbalance.

### Validation and follow-up

W-specific candidates were verified using PCR. Genomic DNA was extracted from the blood of two female and two male White Leghorn chickens using the Qiagen DNeasy kit. Primers were designed for each candidate contig, and amplification was attempted in all four individuals (see Additional file 1 for primer sequences and PCR conditions). If a given contig amplified successfully in both females but not in either male, then it was considered female-specific. Some candidates were verified via PCR in two female and two male Red Junglefowl (UCD 100 Red Jungle Fowl, from M.E. Delany, University of California, Davis). Primer pairs were scored for their ability to produce bands from both female templates that differed from the bands produced from both male templates. Primer pairs with identical results on male and female templates were scored as non-specific. The validation results were used in additional tests of performance. We used independent tests to further investigate the effects of contig length and sample imbalance on the predictive accuracy of our classifier. Validated W-specific candidates will be annotated in Bellott *et al.* in prep.

## Competing interests

The authors declare that they have no competing interests.

## Authors’ contributions

NC prepared DNA samples for sequencing, designed computational method with help from AGC, analyzed all data, and performed some validations. DWB provided the reference genome and performed most validations. DCP managed the BAC-sequencing efforts for the chicken W chromosome. AGC conceived and supervised the study. NC and AGC drafted the manuscript, and DWB and DCP provided critical feedback on the manuscript. All authors read and approved the final manuscript.

## Supplementary Material

Additional file 1 **Table S1.** List of W candidate contigs tested by PCR. Note that contigs that do not have female-specific markers may still be located on the W chromosome. Click here for file
