# Supplementary material for: Identification of avian W-linked contigs by short-read sequencing
Source: BMC Genomics. 2012 May 14;13:183. doi: 10.1186/1471-2164-13-183 (PMC3428670; doi:10.1186/1471-2164-13-183)
Supplement: Additional file 1 — Table S1. List of W candidate contigs tested by PCR. Note that contigs that do not have female-specific markers may still be located on the W chromosome. [file 1471-2164-13-183-S1.pdf]

Supplementary Table 1: List of W candidate contigs tested by PCR. Note that contigs that do not have female-specific markers may still be located on the W chromosome.

Contig name	Female-specific marker?	Left Primer	Right Primer	Annealing temp
Contig197.14	N	TAGGAATGGGATTCAGCCAG	TGGAAAACCGCATTGTGTAA	60°C
Contig197.23	N	ACCCTAAGCCTGACGGATCT	TTCCCATAATGTCACCCCAT	60°C
Contig212.15	N	AATGAGGTCTCACACCAGGG	TAGACACCACGCAAAAGCAG	60°C
Contig212.18	N	ACCTGAAGCAGAGTGGCATT	AGGACAGTGTGTCCCCTCAC	60°C
Contig212.8	N	ACTGCTGCTTTGATTGGCTT	AAGGGTGCTTCATTGTCTGG	60°C
Contig230.11	N	CTGGGTCCATACCTGCACTT	GTCACATTTAGCCAGGCCAT	60°C
Contig243.11	N	GCACATACCCACAACTGCAC	GCGCAAAGTACGCCTCTTAC	60°C
Contig245.12	N	TTGCACATGGATGAGAGTCC	AGGCGTACAGCATCATTCCT	60°C
Contig248.17	N	AGCTTGCACCCTGTCTGAGT	GGGAAACAAGGAAGCTAGGG	60°C
Contig256.6	N	GCTGGCACTCAGGTAAAAGC	ACCTAACGAGCTCAGCAAGC	60°C
Contig260.11	N	GAACCTGTTCCCTAGCACGA	GGCATGTATTCTGGGCAAGT	60°C
Contig269.4	Y	TTACACCTTGAAGGGCTTGC	TTGAGGCTTCATTACTGGGG	60°C
Contig276.6	N	TCGAAATGGTGCTACACAGC	CCCTCAAAATACCTCTGGCA	60°C
Contig282.24	N	GACCTCTGCTTCCTCCTTTG	TGGGACTCTCTGTTGGTTTG	59°C
Contig284.11	N	CCAACTCTAGCTGCGAATCC	TAACAGGTCCTGGAGATGGG	60°C
Contig286.6	N	CTTAAAGCTTGGGAGGAGGG	GTCTGCGAGAGGGGAATAAA	60°C
Contig287.19	N	ACTGCGACCTTTGAAACCAC	AGCTACGCTCGTGTCTTCGT	60°C
Contig291.5	N	TTCCGTAGCGTTTGTTCCTT	ATAGTGACGTTGGCTTTGGG	60°C
Contig297.8	N	ACTCAAGCCAGAGGCGATAA	AGGACAGTGTGTCCCCTCAC	60°C
Contig303.13	N	GCAGGCTGGGTTAGTCAGAG	GTGTGGCAGATCACAGGAGA	60°C
Contig317.11	N	TGTTGTGCCTCATTCCTTTG	GATTTCTGCTGCTCGCTTG	60°C
Contig330.15	N	GCATTGATTGGTCTTGCTCA	GGAATCCTACAGGAACGCAA	60°C
Contig337.3	N	ACAATGGCGAGAGCTCAACT	TACAAAAACCTTTCGGTGGC	60°C
Contig337.4	N	GATGAAATGCAAAGGCTCGT	TGAGATCCTCCTGGCTATGG	60°C
Contig339.4	N	ATGCTGGTTTTCCCAGTGAG	TACAGGGTTTCCCGTGCTAC	60°C
Contig350.1	N	GGAGCCTGACACGTCTTTTC	CTCCGCATAGTATTTCCCGA	60°C
Contig350.3	N	CAGCAAGATGTGGCAAAGAA	TGGGCAGGAGTAGATGGTTC	60°C
Contig353.5	Y	GCTGGGACCACAAGTTCATT	TGCCGACATTTCACATCCTA	60°C
Contig353.6	Y	GTGTTGATCCCTCTGCCATT	GGAAAAGATCCCCCACAAAC	60°C
Contig360.2	N	CAATGGTAGGCTGTCCTGGT	ACAGCGGGTGGTACTTTCAC	60°C
Contig370.2	Y	TGTCTCCAAAGCAACAGACG	GCAGTTGCAATCTGGTGTGT	60°C
Contig379.5	Y	CCAACGCCTCATACGTAAAAA	TGAAGCAGGTAGTTGGGTTG	60°C
Contig380.10	Y	CCGTGTTATAACCCCACTCG	GCAGGGGGTTGGAATTTAAG	60°C
Contig380.12	N	CCCCTTTGCAATCAGAACAT	AACAATTGGGACCACAAAGC	60°C
Contig380.6	Y	AAATCCTTGGACGCCTCTCT	CCACGTGTGCATCTATGGAG	60°C
Contig395.5	Y	CAATTGTCTTCCTTCGGGAG	TCAGCATCCTCAGACACGAC	60°C
Contig395.6	Y	TGATTCATGCCGATTTTTCA	TTCCGAGATCTTTTGAAGGC	60°C
Contig395.7	Y	GGGTTAAACGCACCTCATTC	TACCCCACGATTCAAAAAGC	60°C
Contig399.2	N	CTCCTGAGTCCCACGGATAA	TTCTCTGCATGTGTTGAGGG	60°C
Contig401.2	N	TCAATGGGGTAATAGCAGGC	GTGCTGGAAGTGGACGGTAT	60°C
Contig414.2	N	CCACCGTTTTGTTTCGAGAT	TTGATCCCCCTTAAGAGCCT	60°C
Contig415.2	N	TCAGGGATGAGAGCGTTTCT	GGCTCCACATGACCCATAAC	60°C
Contig416.1	N	GCTCTTCTGAGGACGGACAC	CTCCATCACCACACAAAACG	60°C

Contig419.6	N	TGTCAGTAGGAGAGTAGGAAGTAAGG	CTGGGTCAATGCTGTTCTTG	59°C
Contig432.8	Y	GCAGGGGATTATGTCCTCAC	CTGAGGGTTCCTCATGCAAT	60°C
Contig439.1	N	ACCGGCTGGAAATTTTCTCT	AGATGCAAAATCTGTGCCCT	60°C
Contig451.2	N	CTAGCTCACCAAAACCAGCC	AGCTCCCAAGAGACTGACCA	60°C
Contig455.5	Y	GAGAAGAATCTGCATTGGTGC	TCAACTTGATGAGGGTTCAGTA	60°C
Contig457.3	N	GTAGTGGTCGACCTTCCCAA	ATTAGGCCGTGTGTGTCTCC	60°C
Contig463.1	Y	CCACCTGCCAACCTATCAGT	GCAGAAATTGTCGGTGTCCT	60°C
Contig473.3	Y	TACCTGGAAACCCATACCGA	TGTGCTTCTTGTGCCCTACA	60°C
Contig508.1	N	GCGTGTTGTTGTATGTTGGG	ACTCGTGACCGTAGGCAAAT	60°C
Contig512.3	N	TGCTTTCACAAGTGATTCGG	AGCGCAGAGCTACCTGAAAG	60°C
Contig516.1	Y	CTCCCTGCCCAGATAAATCA	TAGGCATTCCAACGACTTCC	60°C
Contig521.3	N	AGGCCTTGCTTCAAGGTACA	TGATACATCAGGCTTTGGCA	60°C
Contig522.2	Y	ATCTTCGGTCAAGTTGGGTG	CTCCCAAATTTGTGGGTGTT	60°C
Contig522.5	Y	TGGGAGTGCATGTTGTGAAT	CACATGCTCCAGGCATTAGA	60°C
Contig522.6	N	CCTGAATCCATCCCTGAATC	ACAATGCCTTGGACGTGAAT	60°C
Contig528.3	N	ATCAGCCCACCTTAGGGAGT	CCCATTCAAGGACTAGGCAA	60°C
Contig529.1	N	TCCTGCTGATGTCCCACATA	CTTCCCAACCACAAGAGCAT	60°C
Contig558.2	Y	GAAAGACGCAAAATGCAGGT	GCACCTTGGAACAATCAGGT	60°C
Contig563.3	N	AACCCCAGTCACACATAGCC	CACTTGGCTGACCCATACCT	60°C
Contig596.1	N	CGCATTAGGATTTCTTCGGA	CTGCTGCCTACGTGTTGAGA	60°C
Contig611.3	N	TGAGACCCGAATCCCATAGA	TGCACCTAGATCTGTGCCTG	60°C
Contig631.3	N	ATGCTATCCTGCCCAATCAC	GGACCGTATGATGGTGGAAC	60°C
Contig639.1	N	GATACGTGCCCCTTCAGAAA	TTCAGACGTGTAGTGCGTCC	60°C
Contig659.3	N	TGTCGCTACAGCTGTTTTGG	CACTGCAACCGTATGTGAGC	60°C
Contig677.1	N	CTCTGCTCAGTGAACTCCCC	TTCAGCCTTCAAATTACCCG	60°C
Contig684.2	N	AACCAATGGCAAAAGAGACG	CGGTGAGAAGCCGCTAATAA	60°C
Contig697.3	N	CCCTTTTACCACGAGTTCCA	GAATGCATTGGGCACAGAG	60°C
Contig698.1	N	CCATCTCAAGTTGGTGGCTT	AGCTCTCATGCCGTAGCATT	60°C
Contig701.1	Y	CCCTGTTGCACACCTTTCTC	TAACGCAAGAGAGAGCCAAA	60°C
Contig736.1	N	TTTGAGGGACACAAGACCAAC	TGCAACGTTTTTGAAAGTGG	60°C
Contig736.2	N	TTTGAGGGACACAAGACCAAC	TGCAACGTTTTTGAAAGTGG	60°C
Contig744.1	N	CAGGCACATTCACACCAAAC	AGTTCAACGCCTGATGGAGT	60°C
Contig744.2	Y	ATCCAGTGCTCTGCTTGGTT	AATGTTGGCATGTTTGCTCA	60°C
Contig748.1	N	AGCAGTGCAAATTTCAGCCT	AGTTCTCCCTGTGCGAGTGT	60°C
Contig773.7	N	GGAACCGATCCCAGGACTAT	CTATGCAACGGATTTGGGAT	60°C
Contig786.3	N	AATGTCTCTTTGGGGGCTCT	ACATTTTGGGATGCAAAAGC	60°C
Contig788.3	N	GCGTCAAGTGATGTGCTGAT	CGGTGTGTTCTGAAATCCCT	60°C
Contig789.1	N	TCATCCCAAAACACAGCATC	GACACGGAACACGAGACAAG	59°C
Contig804.3	Y	TTGACTCATCTTGCACTGGG	TGCGGAAGCTTGGATTAACT	60°C
Contig823.3	N	GGACGTGAAGTTTGGAGAGC	AATGTGTGGGTTTGGGTCAT	60°C
Contig842.2	N	TCTCCATTGCGAAGAAATCC	ATGCTAGTCATGTCACGGCA	60°C
Contig847.1	N	CTTCCACAGTGACCAGGGTT	CCAGCCACGATAACCAGTCT	60°C
Contig851.1	N	CACTCCAGGCTGAAATGGTC	AGAGTGGGAGCAGCTGGATA	60°C
Contig851.3	N	ACCCCACTTCCATGAGTGAC	GAAACCATCAGGTGTGCTGA	60°C
Contig852.3	N	AGAAGCGTTGTCCAGCATTC	GGTTGGTGAATGTCGCTCTT	60°C
Contig880.3	Y	AGCTTTCTGAAGCGTTGCTC	TTCCTCACTGCGTTCATATTG	60°C
Contig890.1	N	GCTGCCTTGGTTTCTACAGG	ATGGCAAAAGAGAACCGATG	60°C
Contig922.5	N	CCCAGTCACTTCCACTCCAT	TTTGCCCGTTTATTTTCGAC	60°C

Contig925.2	N	GGCCAACTCATGTTACCGTT	AGATTCGTGGAAGATCCGTG	60°C
Contig974.2	N	GGGAGTGATCAGACCGCTAA	ATAGTGACCCATAGCGCTGC	60°C
Contig1003.1	N	TGCTATCACTGTGGCACCTC	CATGGAGATCAACATCGTCG	60°C
Contig1039.3	Y	CAAGTGTGTGCCCTTCCTGT	CTACCTGGCAAACCTAGGCA	60°C
Contig1086.1	N	GGACAGTGTGGCCATTTTCT	TCCAAAGCAGAAGCCCTAAA	60°C
Contig1100.2	N	GGCAAGAGGATGGATCAAGA	CGGGAGGCATAGAAATCAAA	60°C
Contig1103.1	N	CACCAATTCACGCCATACTG	TTTCTTCTCATCTGCACCCC	60°C
Contig1107.2	N	GAAGAGGTTGCTGCACTCGT	TTTCAACGACAGTGATGGGA	60°C
Contig1131.1	N	TGGGTCTTGTCTGTTTGACG	TGGCAAGAATACTGTCAAATCC	60°C
Contig1132.1	N	TGGTGCTGGTTATGGATGAA	TGATCTACGGGTGGTCTTCC	60°C
Contig1151.6	N	GCTTTGGGTGAGCAAAAGAG	GAAAGCGAGGTGCGTAAAAG	60°C
Contig1158.3	N	ATATTTTTCCGTGGCACGTC	TCCTATCACTCGCAGGCTTT	60°C
Contig1180.2	Y	TGAAATCCACCACCCTCAAG	GCCAACCAATAACAGCTTCC	60°C
Contig1194.1	N	CGGCTGTGCAGACTATGAAA	GTCCCCAGGCAGTAGAACAG	60°C
Contig1209.1	N	ACAAAGAGAGCGAAATGGAG	ACCTTGAATAGAGCCTGAAATG	57°C
Contig1220.2	Y	CCGCATAGCTTTTCATCAGTT	AGAGACGAGCCAGAAGATCG	60°C
Contig1231.2	N	TGTTTCTGTGCAGAACCCAG	TCACATGAAAAACTGCCACC	60°C
Contig1240.3	N	GCTATGAAACCGATCCCGTA	GTCCTACTGACTTCGCTGGC	60°C
Contig1249.1	Y	TTCACCCAAATCTTAACAGGG	TGTTGTAACAGGGTGTATCCCA	60°C
Contig1260.1	N	CTGCTTACGGTGGGTGAAAT	TGTAGCGCAGACACATAGCA	60°C
Contig1286.2	N	CATAGAGGTGGGAACCCAAA	GTGGAAGCTGTATGTCCGGT	60°C
Contig1311.3	Y	GCCTTGAACTTGGTGGTTGT	CTACGGCAGCCGAGCTAGTA	60°C
Contig1314.1	Y	CCCACCACACAGGCAATAG	GATGCAAATTCCGCATGTT	60°C
Contig1327.4	N	ATTTCTCAGGGGTGCTGATG	AGTGGGAAGACCATTTGTGC	60°C
Contig1351.5	N	GTGCACATATCCTTCCACCC	GCAGCACTTCGATCATTTCA	60°C
Contig1405.4	Y	GGGAACAATTGCTACCTGGA	CACAAACCGGTTGGGTAAAG	60°C
Contig1409.1	Y	GTGGAGGTTAGACAGCCAGC	CCATGCTAGGCTTCCACAAT	60°C
Contig1420.1	N	GGGAGACCTGATGCCAATAG	TGGATCACAAATGGAGCAAA	60°C
Contig1451.1	N	GAGCTGTTCTGTAGGGCTGG	TTCTGCTTCCCATAAATCCG	60°C
Contig1460.1	N	TGGGTTTCAGGTTCAGGTTC	TCTGGTTGTGTGCTTGTTTG	59°C
Contig1475.2	N	AAAAAGAGGAACAATGCGTTAC	AAATCGTTATCAGCCTGCTC	60°C
Contig1486.1	N	ATCTCCAGGACAGGAGGACC	ATAGGGAAGCCCTCACAGGT	60°C
Contig1506.2	N	AATCTTGGTGGCCTGTTGAC	CGTGACCTCAAGTGGTGCTA	60°C
Contig1510.1	Y	GGGTCTTGCTGGAAAACTGT	GCGTGGAAAATGCCACTATT	60°C
Contig1511.1	Y	TTCACAGATCCTCAGGGAGC	CACAGTAAAGCCCAGAGTTGC	60°C
Contig1515.2	Y	CATGACATCATGCGATAGGC	TAATCGGATTGGCAAACCTC	60°C
Contig1520.1	N	CGTAAGCCAGATTCACCCAT	TAAAAGCCCTCTTCGTGCTG	60°C
Contig1542.2	N	CCTCAGACGTCACAACATGC	AAACCCTCTTTTGTTGCCCT	60°C
Contig1589.2	N	ATTCCCATCTGGTTCAGTCG	AAAAATGGATGCGATTCAGC	60°C
Contig1592.1	N	CAGCGATTGATGGTCTTGAA	CTAGGGAAGGTGCGAACAAG	60°C
Contig1634.3	Y	TGAACCTGGCTCCTCTGAAC	CCTCATTTTGAAGGCAAACC	60°C
Contig1672.1	N	AAATGACAGGCTGTTGGTCC	CTTGCACGTGTGTGTGAATG	60°C
Contig1690.3	N	CCATTCGTAGAACCCCTCAG	CAGCCCACATAGAGTGCGTA	60°C
Contig1739.1	Y	CGTCCAGATGCACAAGCATA	GGTTGGACATATACGCCTGG	60°C
Contig1755.2	N	AATGGGTGTGTGAGAGGAGAG	CTGCTGCCTTTGTGGATTG	60°C
Contig1763.3	Y	CAAGAACCCAAGCAAGGTGT	GGGGTTGTGTAGGGAAAGGT	60°C
Contig1778.2	N	ATGTGGGGTTATATGGCTGC	AACCCCACCTTATGGAGGTC	60°C
Contig1796.2	N	ACTTCACCCTTGCTCACCAT	GCACATGGTGCTATCCAGAA	60°C

Contig1835.1	Y	GTGGCTTCTTGCTCTCATGC	GGAACAGGGGACAAATCTCA	60°C
Contig1835.2	Y	CAAGATCTGGGGATGTTGCT	CAATCCCAGGGTTGAAATGT	60°C
Contig1941.2	N	ACGTGAATTGTGTTCTGTAGCTTG	GCTGGTGGAAATGGTGAATG	61°C
Contig1948.1	N	CTCCAGGCCAATTTGACAAT	TTATCTGGAAGGAGGCAAGC	60°C
Contig1964.5	N	CCTGCTTTTGTGACCCCTAA	ACCCTGATGCCATTTTGTCT	60°C
Contig1974.1	Y	CTCGTCACCATTCCCAGTCT	AGAAATTGAAAAGGAGCGGG	60°C
Contig2014.1	N	CAGAAATTGCAGAAGCTCCC	GCACCTTGTTAGCCTTGCTC	60°C
Contig2033.1	N	CCAGTGCTACTCCATGCTCA	AGCAGGGGATTTTCCTGATT	60°C
Contig2041.1	N	GTAAACTGACCTTCATTCATCTCTC	TTTCTTCCTTTGCCACACTC	58°C
Contig2053.1	N	CCAACAACAGGAAGACCAAATC	TCAGCACGGAGTAGAATGACC	60°C
Contig2178.2	N	AGGGCAAGGGAAGAATCG	ACATTTGACGGCCATTTGAC	60°C
Contig2208.3	Y	GAGTCCATCGAAAGAGACGC	TGCTGGGACATGGTAAGTCA	60°C
Contig2256.1	N	ATTTGGGGACAGGCTTTAGG	AATCCTTTGCTTGGCTACGA	60°C
Contig2319.1	N	AGTCTCTCACCCACCCAATG	GCCACATTTTCCAACCTCTG	60°C
Contig2325.3	N	ATTAAGGGAACACGCCACAC	GAAGCCGGAATAAAGTGGCT	60°C
Contig2561.3	N	CTCGAGCAGCTCCACGTAGT	CTACAAATGTTGGGATGCGA	60°C
Contig2585.1	N	TGTGTGGTGAGACTTCAGCC	CAAATAATTGGGTGTTGCCC	60°C
Contig2603.3	N	TGCACGGCATCATCTAAGAG	CCCCAAAGGGATGAATTTCT	60°C
Contig2615.1	N	GCTCTGATTCAAATGGTGCC	GGCAGTTGCAATAACTGGGT	60°C
Contig2639.1	N	ATTTGAGCTTTGTTGGGGTG	CTGCAGTAAAACCGTGCTTG	60°C
Contig2650.1	Y	CCTTTAACGACCCTCAACCA	CCCATTAATTACAGCCCCCT	60°C
Contig2784.2	Y	CGGATTCACATCCAAACAGA	GACTGCTGCTTTAGATGCTTTG	60°C
Contig2802.2	N	GTCACTGAACCAAACTGGGC	CGTCAAACAGACAAGACCCAT	60°C
Contig2817.2	N	GCGACCTAACAGTCTGCTCC	GCTCCTTTATCCGGTTTCGT	60°C
Contig2838.2	N	GCATTAGTATCCCTGCGAGC	TCAGAGGAAGCCAAGATGCT	60°C
Contig2842.1	N	TAACCACGTGACCCCCTTTA	AGGTGACCCCAATGGAAGAT	60°C
Contig2870.2	N	TATGGGATAATGGCTCCGTC	CAAAATGCCTCCCACAAAGT	60°C
Contig2879.2	N	CATCCCATCAAAGAGACCATC	GAACATCGCAAACCAACCTC	60°C
Contig2883.1	N	TCCAGGGATGCTGTACTTTG	AGCCAAGTCATACCTCCAGC	60°C
Contig2890.1	N	TGCCCCAAGTCTAAAAATGC	CTATGGTCACCCCTTCTCCA	60°C
Contig2952.1	N	TGTCAAGGCTTTGGTTCACA	GTGAGCGGTGTGTTAGAGCA	60°C
Contig2958.1	N	TTTTCACCCCAGAAATGAGC	TAAGCGTACCCAAAACCCAC	60°C
Contig3021.1	N	GCAGGCTGGGTTAGTCAGAG	GTGTGGCAGATCACAGGAGA	60°C
Contig3081.1	N	GAAGCAGCTCTGTCGCTTTTA	GGGGTACTGGGAGCCATTAT	60°C
Contig3118.2	N	AGGACAAAGGCAAAGAGCAG	GAGGAGGAGGAGTGGTTCAG	59°C
Contig3129.1	N	CATTGTACACCCGTCAGCAC	GGACAGCCTTGTGGAGATGA	60°C
Contig3141.3	N	AGTGACGCTTCGTGAGAACC	ACATAAGTGCCCCTCAGCC	60°C
Contig3153.2	N	GCGGGGAAAGATAAAAGAGG	TGAGACACATTAGATGGGGG	60°C
Contig3189.1	Y	ACCCCAATGACCTCGTGTTA	GGAGGACCGGTATTCCAAAG	60°C
Contig3211.1	Y	ATACATGACCGAAGGGACCA	CATGCCAGTGTTGCTGTACC	60°C
Contig3211.2	N	AGTGCACATACCAGAGGGCT	GTGCCGAAATCTCCATCAGT	60°C
Contig3211.3	Y	TTTCCGCTCAGTCAGCTTCT	GCATACGCTTGCCCTGTACT	60°C
Contig3218.1	N	CTCCTCTTTACCTGCGTTGC	ATACTGCATGGGTACAGGGC	60°C
Contig3262.1	Y	CACATGACGCACCTTTATGG	GGCTGTTCTTCCAGGTTTGA	60°C
Contig3296.1	Y	GTGGAGGCATCAGTAGGGCT	TCCCAGTGGATGGATAAGGA	60°C
Contig3298.1	N	TGCCCTGTCTCGCATATACA	CGTCTCGGTTTGGACATTTT	60°C
Contig3318.2	N	GTGAGAGCTAACAAAGCCCG	GAGCGCTAGAACACCCCATA	60°C
Contig3346.2	N	TCTCCGTGGCTTTATTTTGG	ATGTTGGTGCAAAGAGACCC	60°C

Contig3436.4	N	AACAATCCCATAGCCCAGTG	GAGACATTTGGGGGTCTGAA	60°C
Contig3591.2	N	TATAAGGGGCGTGGTCAAAG	TCCACATCCCCTTTCTATGG	60°C
Contig3623.1	Y	GTACATGGGACCAACCCTGT	TGTATCAGGCGTGAGCGTTA	60°C
Contig3641.1	N	TGTTATGAATGGGTAAGGGATG	GGGAGGTAGCAGTCCAGAAG	59°C
Contig3644.1	Y	TTGCCTTATTTTCTGTGCCC	CCGCCTTCTACCGTGTCTAC	60°C
Contig3707.2	N	TTTCTCTACCCTTCAACCCA	TTAAAAGCACGTTCACGCAC	60°C
Contig3734.1	N	CATTGAGCCAACCAGTTGAA	GGTTGGCTGTAAGGACAGGA	60°C
Contig3835.2	N	AACTTGGATCAGTGGTTGGC	TGAGCTCTGGTTTGGGACTT	60°C
Contig3951.1	N	TAGGGTGATGGGAGGTGAAA	CCATACAAGCCTCCCCTACA	60°C
Contig3971.2	Y	GCAGCCCTTCCTGAACATTA	CCAGATGTTAATTCCGTGGG	60°C
Contig4134.1	N	GATTTCTTCGGAGGCTTGC	TGTAGGCCTTGGGCTTATTG	60°C
Contig4139.1	N	TTTAAAGCCTGTGTCCACCC	ACAGGTGCGACCCTAATGAC	60°C
Contig4166.1	N	AGCAGAAGGCGACATTTTGT	GCTGAAGCTGGTACAGGGAA	60°C
Contig4220.1	N	AATTCTTTACCCACGTGCAA	TTTCCTTGAGGTGTGAGCCT	60°C
Contig4244.2	N	GCCCCATTCCAAACCTTATT	CATATCTGCCCCAAATCCAC	60°C
Contig4303.1	N	CTCATAACAGCCCCTCGAAC	TTTTGGGTGTTTTGGGGTAG	60°C
Contig4350.3	Y	CGGCGAGTGGGTGTATCTAT	CAGCCACCTTCAACATCAAC	60°C
Contig4351.2	Y	AGGCACCCGTATTCACAAAA	CCCCATAAGGGGCAAACTAT	60°C
Contig4483.2	N	GTGCTAAGGGGATTTCGTGA	TCACGGGTCTTTAGGGTCAG	60°C
Contig4605.2	Y	AAAAATCCTTTTGAGGCAGTCA	GCTTTACATTTTGGCTCCCA	60°C
Contig4688.1	N	TTGTTCCTGAGGGGATTGTC	GCACGGTGAGGGAATAAAAA	60°C
Contig4707.1	N	AAATGTCGCGAAAGAAAAGC	CACTGGGTCTGGCGAACTAT	60°C
Contig4708.1	N	CACGATTCAGAGGTCAAGCA	ATGAGTTTTGCACCCTCCAG	60°C
Contig4746.1	N	CAAAGGCTTTTGGGTAGCAG	CTGTAGCTGCGTGCATTCAT	60°C
Contig4748.1	N	CAAAGGCTTTTGGGTAGCAG	CTGTAGCTGCGTGCATTCAT	60°C
Contig4915.3	N	AATGCAACGCAGACCTAACC	GGAGCTTGGAAGACTGCGTA	60°C
Contig4937.1	N	ACATGTGCCATACACACGCT	GTCTGCGTGGTAAAGGGGT	60°C
Contig4944.1	N	ACCTGCACAATTTCCCATCT	GCCACAATGTATGGCAAAGA	60°C
Contig5029.1	N	GAACGGGCTTAAAGGAAAGG	AGCTGCCCGTGAAAACTAGA	60°C
Contig5053.2	N	GCGTGTTGTTGTATGTTGGG	ACTCGTGACCGTAGGCAAAT	60°C
Contig5100.1	N	ACGCTGTTTTGTCCTAACGC	ACTTCAGTTCAAACACCCCG	60°C
Contig5216.2	N	TATTTGTTGCCATGTGGGTG	ATATGGACCCAGAGCACGTC	60°C
Contig5266.2	N	GGGAGCCCAGTTTACCTCAT	GAGGCCCAGTAAGAGCTGTG	60°C
Contig5288.1	N	TTTGTGGCAAGACACGGTAG	CCCGCCCTACAATTATGATG	60°C
Contig5418.1	N	TCCTGCTGATGTCCCACATA	CTTCCCAACCACAAGAGCAT	60°C
Contig5696.1	N	CCTGTTCCTGTTCCTGTTCC	GAATCCCTTGTGCCTGAAGA	60°C
Contig5780.2	N	GCTGGTTGGGTTGTGGTC	AAGTACCGCCCCATATCACA	60°C
Contig5876.1	N	CTTGGGTTTTGGCTGATGAT	AAAATCCATAAGGGGTTGCC	60°C
Contig6113.2	N	CTCATCCCGCTCCAAATC	TGCTATTACCCTACAGTGGCTTC	59°C
Contig6373.1	N	ATACCTGCTCTCCCCTCCAA	AGGGGTTTATGGACCCAGAC	60°C
Contig6408.2	Y	GACTCTCCTCCACCATCTTCTG	ATTCACCACACTGGGCTTTG	61°C
Contig6502.1	N	AGGTCACCGACAGCGTAAAC	AACCCACAATCCCATCTGAA	60°C
Contig6513.2	N	GTTGATAGGGACCTGACGGA	ACACTTGTAGCCGTGGGAAG	60°C
Contig6764.1	N	GACCGCTCACTCACTGTCCT	TGTCATTGCTTGGGTGATGT	60°C
Contig6895.1	Y	ATACCAGTTGCAGGGCTACG	CAAGACCTTTCTGCTCCCAT	60°C
Contig6910.1	N	CCCTTTTACCACGAGTTCCA	GAATGCATTGGGCACAGAG	60°C
Contig7121.1	N	ACGGGTATGAGGTTCAATGC	GCACCTTAATGCCTCTCCAC	60°C
Contig7339.1	N	CTGTCTGCAGAATGAGCCTG	CAAGTGGAGATGGGACCTGT	60°C

Contig7456.2	N	CCCGCACCAATATAAGCATT	GACCCATCATGGTGACAGTG	60°C
Contig7563.2	N	AGATGTTGGGGGCTCTTAGG	AGTATCGCCCCATTGACAAC	60°C
Contig7578.1	N	ACTTTGGAGACCCCTCACAC	GGGGTGTTGGGAGGATCTAT	60°C
Contig7896.1	N	GGAGGGATTTGGTTTGGTTT	GCCCCACAACCTTCCTCTAT	60°C
Contig7947.2	N	CGAGGAGGAGGAAGATGATTG	CCACGCCCACAGTAGAAGAG	61°C
Contig8091.1	N	CCACCTGTGAGATCTTTGGC	TCTTGAAATAGGTGGGGTCG	60°C
Contig8209.1	N	AAGGGAAGCAGATGGAAGTG	GGCTGAGGAAGGGACAATC	59°C
Contig8227.2	N	ACTGGCCCCACCAACCTAC	GAAGCCCATGCCCATCAC	62°C
Contig8239.1	N	TGCTACCTAGCTGCCTCCTC	ATTGACTGCTGATTGCTCCA	60°C
Contig8478.1	N	GAATCTCAACAAAGGCCATCA	TCAGACGTGTGGCCAGAATA	60°C
Contig8600.1	N	CTCAGTGGAGAGGGCAAAAG	GACCTAATCAAGCCCAGCAA	60°C
Contig8693.1	N	CATCATCCCTCCCATCTTG	CTAAGCATTCCCTTTCACAATC	58°C
Contig8762.1	N	CTGGATGACCTGAAACGTGA	GATGGCATTCAAGGGTGAGT	60°C
Contig9315.1	Y	AGCAGTTTATGGGGGAACAG	AACTGCATGCATGTATGTCTCC	60°C
Contig10123.1	N	CACCCAGTTCTCAAGGCTTC	ATGGGGAAAGGTTTCGAAGT	60°C
Contig10132.1	N	ACACATCGCCTCATCTTTCC	GTTTCTTCCCAGCACCTCAC	60°C
Contig10152.1	N	AAGAGAGCGTTTGGGTAGGG	GGGTTTGGGTTGGAATCAC	60°C
Contig10235.1	N	CACCCAGTTCTCAAGGCTTC	ATGGGGAAAGGTTTCGAAGT	60°C
Contig10549.1	N	GAATGGGCCTTTTCTTGTCA	TTTGAGAGGCATACACAGCG	60°C
Contig10935.1	N	GGCAAGAGGATGGATCAAGA	CGGGAGGCATAGAAATCAAA	60°C
Contig11104.1	N	ATTTAAGGCCCAGAATCCCA	GCAACCAAGTGGACGTTTTT	60°C
Contig11194.1	N	ATGAAACCCTGTGTCCTCCA	TGATGAAATCTCCCTCCCAT	60°C
Contig11319.1	N	AAATGACCCCAAAAACCACA	CCATTCTCTCCGTCAACCTC	60°C
Contig11338.1	Y	CCTAAGTTTGTTCCCGCCTT	ACTTTCCACCATTGCACAGA	60°C
Contig11523.1	N	ATGAGTTGGCCGATGACTGT	GGTATGATCACTCACGCCCT	60°C
Contig11635.1	N	TCAGTTTCCAAGTCCGTTCC	CCCTGGGCAGATTTGAAGTA	60°C
Contig11638.1	N	TCCCTGCCATTCATTTATCTTC	AATCAGTCATCCTGCCAACC	60°C
Contig11691.1	Y	ACGTGCAGAGTAGCTTGCCT	CCCCATTCCCCATAAACTCT	60°C
Contig11727.2	N	AGCGTTGAGTAATGGTTGGG	GAGGTTGAGGCCACTGAATC	60°C
Contig11747.1	Y	AGATCAACTTCAGGGTGGCA	CCTCCTTGCAGATAACGTGG	60°C
Contig11847.1	N	CTGTGTGAAAGGTGGTTCCC	GATCCCAGGACACATAGCGT	60°C
Contig11950.1	N	AGAATGTGGAAAAACCCGC	GGCTCTTCTTGAAAGGTTCG	60°C
Contig11957.1	N	AGCAGATGAGTTCCACCAGG	GGCATGAGATATGTGGGGAC	60°C
Contig12399.1	N	GACCCACAGATCCCAGTTTG	AAAACGTGGTATGGGGCAC	60°C
Contig13219.1	N	AAGGGATCACAGCAACCAAC	TCCCTTTTTCAAAGCGTTTCT	60°C
Contig13772.1	N	GATGCCAGAAGCGTTTGC	GAGAGGGGATGACTTCGACA	60°C
Contig13865.1	N	GCATCTGGAGACACCTGGAT	GGAAAGCCTGGGTAATGGTT	60°C
Contig14186.1	N	TCCCTGCAGCACTGTAAATG	ATACTCCCGAGCATGAGGGT	60°C
Contig14188.1	N	GGAGCATTCATAGCCACTAAAAC	CCACTTCACAGGGACTTCTTC	59°C
Contig14260.1	N	TGTTGTGCACGTGGATTCTT	CCGAGAAGTCAGCCTAAACG	60°C
Contig14439.1	N	GAGAGGTGAAGGTGGTGCTG	AGGAGGACGAGGTGGTGTTG	62°C
Contig14772.1	N	CTGACGGAAATCTATGGGGA	TTGCCATAAGACTACGGGCT	60°C
Contig15203.1	N	CTCGTTAGCCTAAACCCACG	CCGGGGGATTTTAATAGAGG	60°C
Contig15501.1	N	GGTCCTTTCTTCTGTTGCTTG	ACACACTTCACCTTGTCTCTAATG	58°C
Contig15774.1	N	CCCATGGCCTTTTAGAGGAT	GGTATGAATCCATGCCCTGT	60°C
Contig15996.1	N	GGCCCTGGTAGTACTCCTCC	AGTGGTGATGGATGCACAAA	60°C
Contig16112.1	N	ATGGAGGGGGTTGAGGACT	CTGGCAATTGCTACGGAACT	60°C
Contig16657.1	Y	CCGAGGCAGAAAATAGTGGA	AGCCTTGAGAATCGTGCAAT	60°C

Contig16713.1	N	GCTGTGGTGGGAAGTGATG	TTGCCTCTTTGACCTCTTGG	60°C
Contig16781.1	N	AAGCGGGGTGGTGTGTATC	CAAGCAACGGGATGGAAG	60°C
Contig16914.1	N	ACAGGGTAAGCAGCCTCACA	CATGGATCCTTGTGCTCGTA	60°C
Contig17240.1	Y	TGTGTCCGAAGAAGATGCTG	GGGCAAAGAACCAATGAGG	60°C
Contig17520.1	N	TTCCCGCCCGTATTTGTA	GGCCAGGGAGTTAACACTGA	60°C
Contig17660.1	N	GCATTGATTGGTCTTGCTCA	GGGATGGAAGGGAATCCTAC	60°C
Contig18173.1	N	TTCTGCACAGACCCACTCAC	GCACATCGTATCCGATCACA	60°C
Contig18661.1	N	TGCTGCACCGTCAGATTTTA	CAGTCCATCTGCGTGCTTTA	60°C
Contig18681.1	N	TTTAAAGTGTGTTGCGCTGG	GTAAAGATAAGCCCTCCCCC	60°C
Contig19045.1	N	TCTAATGGATGGGGTAAGTGTG	TGGTGTTCGGTTGAAATCTG	59°C
Contig19184.1	Y	TCTTGGACCCTTAATTGCTTG	TGCCATGCTTATGAGTCAGC	60°C
Contig19387.1	N	TTTGGGGTTTTAGGGGTCTT	CCCCAAAATGACCGAAAATA	60°C
Contig19481.1	N	CTCCAGGCCAATTTGACAAT	TTATCTGGAAGGAGGCAAGC	60°C
Contig19668.1	N	AGACGAATGTGAAACGGAGC	AAACTGCACTCCGCTGAAAT	60°C
Contig19853.1	N	TCAGTTCACGTGTCCTTTGG	GCCAGTAATGAGCTGCCTCT	60°C
Contig20120.1	N	GCCTTCTGTGCACTTTATGG	GATTGGCCGCTACCTCAGA	60°C
Contig20335.1	N	CCATCTATGGGCAATCCAAC	ACTCGCTGGTGACTCAGCTC	60°C
Contig20734.1	N	AGAGCTGTTTCCAATCTGCG	CCTGTTCAATTAACGGCTGG	60°C
Contig21126.1	N	AGAGCCCTTACAGGGTCCAT	ACCCCATAAAGTCCCCTGAC	60°C
Contig21340.1	N	CCCCAAGGGACCATTTATTT	AGGAGGGGCAACTTTTTCTT	60°C
Contig21666.1	N	GGAAAGGGACAGGGTAGGAC	TGTGGCTCTACAAAACCCCT	60°C
Contig21669.1	N	TACGCTCCCTGCTATCTTCC	GCCCTGCTGTTCCTTATTG	59°C
Contig21870.1	N	CCTGTGTGCTGATTGTATCCTG	CTGTGGCTCGGACTGTTCTC	61°C
Contig21949.1	N	ACTTGAGCAGCTGTCGTGGT	ACTCCAATCTCGACCCACAC	60°C
Contig21953.1	N	GGAGCGGTTGGTTTGTCTG	CGCCTTGGGTTTGTTTCTG	62°C
Contig22089.1	N	ATTTGAGTTTGGCTGCAAGG	GCCCATGTGACTAAACAGCA	60°C
Contig22504.1	N	TTATTATCCCCACCGGGAAG	CGATTAAAAGTGCAGGGAGC	60°C
Contig23065.1	N	TTATGGAAATTGGAGCCAGG	TTTGAAAGCATTCTGCACCT	60°C
Contig23455.1	N	ATTAAGGGAACACGCCACAC	GAAGCCGGAATAAAGTGGCT	60°C
Contig23497.1	N	GCAGATAGGAGGTGGAGGAG	TCAGAGAGGTGAGGTTGTGG	59°C
Contig23624.1	N	AGAAGGTTGGGTGAGTGCTG	CATCTGGGATTTGTTGAATGG	60°C
Contig23699.1	N	GTGCCTCATTGCACAAACAC	TAATGCTTGTGCATCTTCCG	60°C
